# Research Progress on Anti‐Inflammatory Adipokine SFRP5‐Mediated Lipid Metabolism and Its Potential Role in Neural Development

**DOI:** 10.1002/iid3.70469

**Published:** 2026-06-21

**Authors:** Qianfang Jia, Zihan Li, Tianyi Yang, Weihua Yang, Wei Chi

**Affiliations:** ^1^ The First Clinical Medical College Zhejiang Chinese Medical University Hangzhou China; ^2^ The First Affiliated Hospital Zhejiang Chinese Medical University Hangzhou China; ^3^ The First Clinical College Henan Medical University Xinxiang China; ^4^ The First Affiliated Hospital Henan Medical University Xinxiang China; ^5^ Shenzhen Eye Hospital, Shenzhen Eye Medical Center Southern Medical University Shenzhen China

**Keywords:** lipid metabolism, neural development, SFRP5

## Abstract

**Objective:**

To systematically review the structural features of secreted frizzled‑related protein 5 (SFRP5) and its dual regulation of canonical/non‑canonical Wnt signaling, analyze its association with metabolic disorders, and specifically explore the role and mechanisms of the SFRP5–lipid metabolism axis in neural and optic nerve development.

**Methods:**

A systematic literature search was performed to review the molecular structure of SFRP5, its regulation of Wnt pathways, and its relationship with metabolic dysregulation. The mechanisms by which SFRP5 modulates the microglia/astrocyte‑mediated neuroimmune microenvironment, myelination, synaptic plasticity, and neuronal mitochondrial energy homeostasis were analyzed, and current therapeutic strategies targeting the SFRP5 network were summarized.

**Results:**

SFRP5 participates in normal central nervous system development by shaping the neuroimmune microenvironment, promoting myelination, regulating synaptic plasticity, and maintaining mitochondrial energy balance. Under obese and diabetic conditions, downregulation of SFRP5 leads to overactivation of Wnt5a/JNK signaling, resulting in lipid metabolic disturbances and neuroinflammation. These changes share common pathological features with neurodevelopmental disorders such as autism spectrum disorder, intellectual disability, optic nerve hypoplasia, and retinal vascular dysplasia. The SFRP5–lipid metabolism axis plays a critical role in neural and optic nerve development, and its dysregulation underlies the neuropathology associated with metabolic diseases. Therapeutic interventions explored to date—including recombinant protein, gene therapy, small‑molecule activators, and acupuncture—have shown promising potential.

**Conclusion:**

The SFRP5–lipid metabolism axis represents a key link connecting metabolic disorders with neurodevelopmental abnormalities. Future research should focus on spatiotemporal specificity at single‑cell resolution, elucidation of gene–environment interactions, and the development of efficient central nervous system delivery systems, thereby providing new avenues for the prevention and treatment of neural and optic nerve developmental abnormalities.

## 1 Introduction

Neurodevelopment is the process of brain structural and functional maturation that extends from the fetal period through adolescence, with critical period plasticity as its core feature. During this process, the brain is under precise molecular regulation and highly dependent on experiential input to establish and optimize functional connectivity, while also being exquisitely sensitive to the external environment, where factors such as nutritional status, infection, and metabolic conditions can adversely affect this process [1]. When the course of neurodevelopment is disrupted, a spectrum of adverse outcomes may ensue, manifesting as impaired neuronal migration, reduced synaptic density, and neurotransmitter system dysregulation [2]. In severe cases, some individuals may meet clinical diagnostic criteria, presenting as intellectual developmental disorder, autism spectrum disorder (ASD), and attention‐deficit/hyperactivity disorder, with a core feature of early‐onset and persistent impairment in social interaction, cognition, or learning abilities [3]. Notably, neurodevelopmental abnormalities frequently involve the visual system. The prevalence of strabismus, amblyopia, and refractive errors is significantly higher in children with ASD compared to the general population, suggesting that clinically diagnosed neurodevelopmental conditions and visual system development may share common abnormalities in early neurodevelopmental mechanisms [4]. The pathogenesis of neural developmental abnormalities involves complex interactions among genetic, environmental, and metabolic factors. In recent years, the role of inflammatory processes in the pathological mechanisms of neural developmental abnormalities has become increasingly prominent. Factors such as maternal infection during pregnancy, autoimmune diseases, or metabolic disorders can trigger inflammatory responses and have been identified as significant risk factors for adverse neurodevelopmental outcomes in offspring. Furthermore, the relationship between metabolic disturbances and abnormal neural development has garnered increasing attention, with lipid metabolism imbalance emerging as a key link connecting metabolic diseases and aberrant neural development, thus becoming a research hotspot.

Secreted frizzled‐related protein 5 (SFRP5) is a novel anti‐inflammatory adipokine. As a key molecule linking inflammation and metabolism, it has received widespread attention in recent years. SFRP5 exerts anti‐inflammatory effects by antagonizing the Wnt5a/JNK signaling pathway, participates in the regulation of lipid metabolism as an adipokine, and is also involved in nervous system development and function maintenance [5]. This article reviews the latest research progress on SFRP5‐mediated lipid metabolism pathways in neural development and related abnormalities and explores its pathological mechanisms and translational application prospects.

## 2 Molecular Characteristics and Biological Functions of SFRP5

### 2.1 Molecular Structure and Expression Characteristics

**Figure 1:**
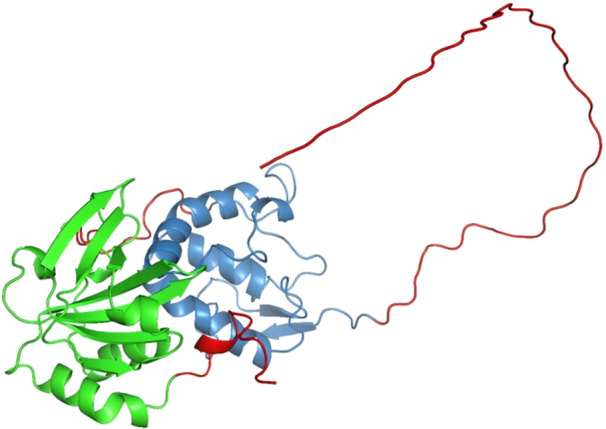
Predicted three‐dimensional structure of the SFRP5 protein. Sfrp5 protein contains two core functional domains: the blue region represents the Cysteine‐Rich Domain (CRD), and the green region represents the N‐terminal signal peptide domain, also known as the Netrin‐related motif (NTR).

SFRP5 belongs to the secreted frizzled‐related protein (SFRP) family. It is composed of 317 amino acid residues, with a molecular weight of approximately 30–40 kDa, and is encoded by a gene located on chromosome 10q24.1. We obtained the latest predicted three‐dimensional structure of the SFRP5 protein from the AlphaFold database (https://alphafold.com/) [6, 7], as shown in Figure 1. Its structural features include two key functional domains: a domain resembling the netrin family, and an N‐terminal signal peptide domain that mediates protein secretion. Additionally, a cysteine‐rich domain (CRD) confers affinity for binding to Wnt ligands, thereby modulating the Wnt signaling pathway and participating in developmental regulation and adult homeostasis [8].

SFRP5 was initially identified in retinal pigment epithelial cells and pancreatic epithelial cells. In 2010, Ouchi et al. [9] first reported high expression of SFRP5 in white adipose tissue, revealing its novel function as an adipokine. In addition to adipose tissue, SFRP5 is expressed in various other tissues and organs, including the central nervous system (such as the hypothalamus), liver, and placenta, suggesting its involvement in the regulation of diverse physiological processes [10].

Notably, the *Sfrp5* gene harbors multiple mutation sites that significantly impact its function. For example, rs10748709 is located in the promoter region of the *Sfrp5* gene and shows an association trend with glucose metabolism, suggesting that this site may influence gene transcription efficiency. Clinical studies [11] have shown that SFRP5 is a key regulator of insulin sensitivity, and its expression levels are finely regulated by genetic background. Variants in the promoter region may contribute to inter‐individual differences in glucose metabolism by affecting *Sfrp5* expression levels. Another key mutation site, rs7072751, is situated in the regulatory region of the gene, where the minor allele is significantly associated with increased total abdominal fat and subcutaneous fat in obese males. Researchers hypothesize that this variant may exert its effect by altering transcription factor binding in the surrounding sequence [12].

### 2.2 Regulatory Mechanism of the Wnt Signaling Pathway

The discovery of the *Wingless‐type MMTV integration site family* (*Wnt*) gene family dates back to 1982, when its first member, *Int1*, was identified as an integration site for mouse mammary tumor virus [13]. Due to the homology between this gene and the *wingless* gene in Drosophila, they were collectively named *Wnt* genes [14]. *Wnt* genes encode the Wnt protein family, which initiates complex intracellular signaling cascades by binding to the Frizzled receptor family on the cell membrane, ultimately regulating the transcriptional activation or post‐transcriptional translation of target genes [15]. Wnt signaling pathways include the canonical Wnt/β‐catenin signaling pathway and non‐canonical pathways such as Wnt/Ca^2+^ and Wnt/JNK. The initiation of Wnt signaling depends on the binding of extracellular Wnt ligands to the transmembrane receptor Frizzled. As an endogenous antagonist, SFRP5 possesses a cysteine‐rich domain that is highly homologous to the Frizzled receptor, enabling it to competitively bind Wnt ligands and thereby block pathway activation.

In the canonical Wnt/β‐catenin pathway, the binding of Wnt to the Frizzled receptor is the primary step in initiating signal transduction. When SFRP5 competitively binds Wnt ligands, the Frizzled receptor cannot be activated, thus failing to recruit cytoplasmic Disheveled and initiate downstream signal transduction [16]. Under this condition, the degradation complex composed of Axin, APC, and GSK3β remains active, continuously phosphorylating β‐catenin in the cytoplasm. Phosphorylated β‐catenin is ubiquitinated and degraded via the proteasomal pathway, preventing its stable accumulation in the cytoplasm. As free β‐catenin levels remain low, its translocation to the nucleus is blocked, preventing it from binding to the TCF/LEF family of transcription factors to form a transcriptional complex, thereby inhibiting the transcriptional activation of downstream target genes.

**Figure 2:**
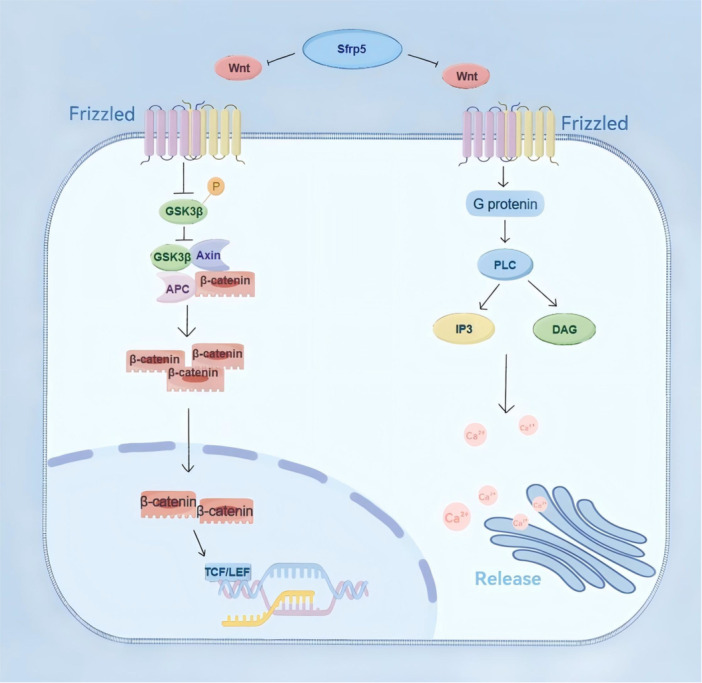
Mechanism of SFRP5/Wnt interaction. Abbreviations: Wnt, Wingless‐type MMTV integration site family; Sfrp5, Secreted frizzled‐related protein 5; Frizzled, Frizzled receptor; GSK3β, Glycogen synthase kinase‐3β; Axin, Axin; APC, Adenomatous polyposis coli protein; β‐catenin, βcatenin; TCF/LEF, T‐cell factor/lymphoid enhancer factor; G protein, Guanine nucleotide‐binding protein; PLC, Phospholipase C; IP3, Inositol trisphosphate; DAG, Diacylglycerol; Ca^2+^, Calcium ion.

In non‐canonical Wnt pathways, SFRP5 competitively binds ligands such as Wnt5a, blocking the coupling and activation of G proteins with the Frizzled receptor. Under normal conditions, binding of Wnt5a to the Frizzled receptor activates associated G proteins, which in turn activate phospholipase C (PLC). Activated PLC hydrolyzes the membrane phospholipid phosphatidylinositol 4,5‐bisphosphate (PIP_2_), generating the two important second messengers inositol trisphosphate (IP_3_) and diacylglycerol (DAG). IP_3_ acts on IP_3_ receptors on the endoplasmic reticulum to promote the release of intracellular calcium ions, while DAG activates protein kinase C (PKC), initiating a cascade of downstream signaling molecules. By blocking the binding of Wnt5a to Frizzled, SFRP5 prevents these signaling events from occurring, thereby inhibiting the activation of non‐canonical pathways (as shown in Figure 2).

In addition to the Wnt/Ca^2+^ pathway, non‐canonical Wnt signaling also exerts important biological functions by activating the c‐Jun N‐terminal kinase (JNK) cascade. JNK is a member of the mitogen‐activated protein kinase (MAPK) family and is encoded by three mammalian genes—*Mapk8 (Jnk1)*, *Mapk9 (Jnk2)*, and *Mapk10 (Jnk3)*—which collectively generate ten different isoforms [17]. Among these, JNK1 and JNK2 are ubiquitously expressed, whereas JNK3 is primarily expressed in the brain, heart, and testes [18]. Upon phosphorylation and activation by upstream MAPK kinases (MKK4/7), JNK acts on various substrates, including transcription factors, cytoskeletal proteins, and membrane receptors, thereby regulating processes such as cytoskeletal remodeling, cell migration, apoptosis, and inflammatory responses [19].

**Figure 3:**
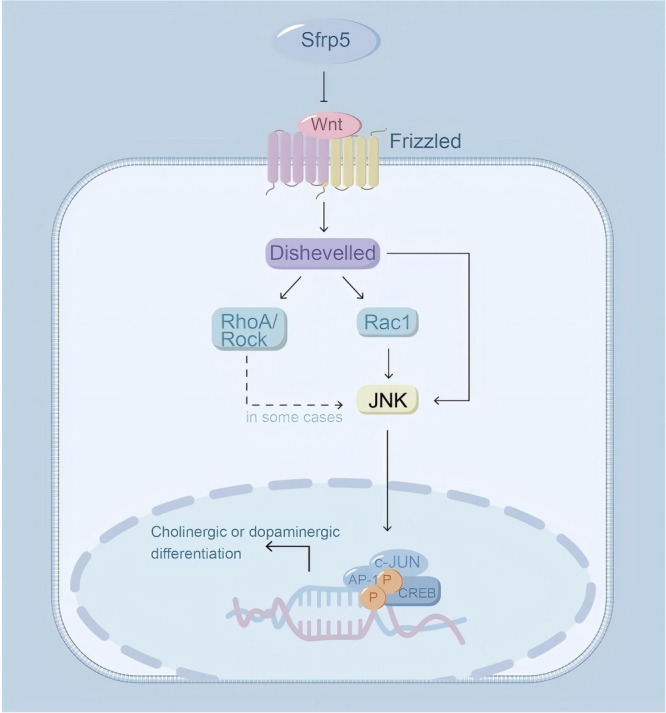
Mechanism of SFRP5/Wnt/JNK interaction. Abbreviations: Wnt, Wingless‐type MMTV integration site family; Sfrp5, Secreted frizzled‐related protein 5; Frizzled, Frizzled receptor; Disheveled, Disheveled; RhoA, Ras homolog family member A; Rock, Rho‐associated protein kinase; Rac1, Rac family small GTPase 1; JNK, c‐Jun N‐terminal kinase; c‐JUN, c‐Jun protein; AP‐1, Activator protein 1; CREB, cAMP response element‐binding protein.

In the non‐canonical Wnt/JNK pathway, binding of Wnt5a to the Frizzled receptor activates the small G proteins (RhoA and Rac1) via Disheveled, which in turn initiates a kinase cascade mediated by MLK and MKK4/7, ultimately leading to JNK phosphorylation and activation. Activated JNK translocates to the nucleus, where it regulates the activity of transcription factors such as c‐Jun and AP‐1, thereby influencing gene expression programs related to neuroinflammation, synaptic plasticity, and neuronal differentiation. As an endogenous antagonist of Wnt signaling, SFRP5 has been identified as an anti‐inflammatory adipokine. Through its cysteine‐rich domain (CRD), SFRP5 competitively binds Wnt5a, blocking its interaction with the Frizzled receptor and thereby inhibiting JNK activation, as shown in Figure 3. Given the critical role of JNK signaling in neural stem cell maintenance, neuronal differentiation, and the regulation of neuroinflammation, the ability of SFRP5 to suppress the non‐canonical Wnt/JNK pathway may represent one of the key mechanisms underlying its protective role in neurodevelopment.

In summary, SFRP5 competitively binds Wnt ligands and simultaneously inhibits the activation of both the canonical Wnt/β‐catenin pathway and non‐canonical Wnt pathways. This dual inhibitory effect establishes SFRP5 as a key negative regulator of Wnt signaling, which may ultimately lead to functional abnormalities resulting from metabolic disturbances, affecting processes such as cell proliferation, differentiation, and migration.

## 3 Anti‐Inflammatory Function of SFRP5

The anti‐inflammatory function of SFRP5 is primarily exerted through antagonizing the Wnt5a/JNK signaling pathway, thereby conferring protective effects in various inflammatory diseases. Existing studies have revealed the regulatory role of SFRP5 across different pathophysiological scenarios.

In acute inflammatory diseases, the protective effect of SFRP5 is particularly prominent. Myocardial ischemia/reperfusion injury refers to the excessive inflammatory response triggered upon blood flow restoration, which further exacerbates tissue damage [20, 21, 22], with macrophage‐mediated inflammation playing a critical role [23]. Studies have found that SFRP5 knockout mice exhibited increased infiltration of Wnt5a‐positive macrophages in the infarcted area following cardiac ischemia/reperfusion injury, accompanied by enhanced expression of inflammatory cytokines and chemokine genes [24]. In contrast, although wild‐type mice also developed cardiac injury and inflammatory responses, the severity was significantly lower than that observed in knockout mice. This indicates that SFRP5 suppresses macrophage‐mediated inflammatory responses by antagonizing the Wnt5a/JNK signaling pathway, thereby exerting a protective effect on the heart. Similarly, sepsis is a systemic inflammatory response syndrome caused by severe infection, which can lead to multiple organ failure [25, 26]. Hohlstein et al. found that serum SFRP5 levels in patients with sepsis were significantly lower than those in healthy individuals, and the degree of reduction was closely correlated with disease severity [27]. This finding suggests that decreased SFRP5 levels may lead to enhanced Wnt5a‐mediated pro‐inflammatory signaling, thereby exacerbating the systemic inflammatory state. Notably, SFRP5 levels in sepsis patients recovered after the first week of treatment, implying that alleviation of the inflammatory state may be associated with restoration of the SFRP5/Wnt5a system balance.

In local chronic inflammatory diseases, SFRP5 also plays a critical regulatory role. Periodontitis is a local chronic inflammatory disease caused by bacterial infection, leading to periodontal tissue destruction and alveolar bone resorption [28]. SFRP5 plays a key protective role in regulating local inflammatory responses. In healthy gingival tissue, SFRP5 expression is predominant, whereas in periodontitis lesions, Wnt5a expression is significantly increased and SFRP5 expression is relatively decreased. Clinical studies have shown that serum SFRP5 levels in patients with severe periodontitis accompanied by periodontal attachment loss are significantly lower than those in healthy controls or patients without periodontal attachment loss [29]. The anti‐inflammatory effect of SFRP5 has been further validated in a mouse model of periodontitis, where local administration of recombinant SFRP5 protein significantly inhibited gingival tissue inflammation, reduced alveolar bone resorption, and decreased the number of osteoclasts [30]. Similarly, osteoarthritis is a chronic joint disease characterized primarily by degenerative changes in articular cartilage, with inflammatory responses playing an important role in disease progression [31, 32]. SFRP5 exerts a protective role during the disease course, as extracellular vesicles released by neutrophils upregulate SFRP5 expression in chondrocytes, thereby inhibiting cartilage catabolism. Animal experiments further confirmed that intra‐articular injection of recombinant SFRP5 effectively attenuated articular cartilage degeneration in experimental osteoarthritis [33]. In rheumatoid arthritis, SFRP5 also exhibits potent anti‐inflammatory properties. Studies have found that overexpression of SFRP5 in rheumatoid arthritis synovial fibroblasts effectively inhibited the production of various pro‐inflammatory cytokines and chemokines, while simultaneously reducing the expression of cyclooxygenase‐2 and matrix metalloproteinase‐9 MMP‐9. Conversely, SFRP5 knockdown exacerbated the production of these inflammatory mediators [34].

In summary, SFRP5 exerts anti‐inflammatory effects in a variety of inflammatory diseases primarily by antagonizing the Wnt5a/JNK signaling pathway. From acute inflammation to local chronic inflammation, SFRP5 expression levels are inversely correlated with disease severity, and its protective mechanisms manifest as inhibition of macrophage infiltration, reduction of pro‐inflammatory cytokine production, and other aspects. Notably, in patients with sepsis, SFRP5 levels recover as the condition improves, indicating a dynamic association with the inflammatory state. These findings collectively highlight the critical role of SFRP5 in the regulation of inflammation.

## 4 Regulation of Energy Metabolism by SFRP5

### 4.1 SFRP5‐Mediated Regulation of Glucose Metabolism

SFRP5 primarily antagonizes the non‐canonical Wnt5a pathway, thereby inhibiting downstream JNK activation, reducing the production of inflammatory cytokines, and improving the phosphorylation status of *insulin receptor substrate‐1* (*IRS‐1*), which enhances insulin signaling and maintains glucose homeostasis [35, 36]. Population‐based studies have provided multi‐level clinical evidence for the above mechanisms. One study in adolescents found that low plasma SFRP5 levels were an independent risk factor for elevated fasting blood glucose, and this association remained significant after adjusting for multiple confounding factors [37]. Another study in an elderly population also found that SFRP5 levels were negatively correlated with fasting blood glucose, indicating that this association exists across different age groups [38]. Furthermore, in patients with heart failure and type 2 diabetes, SFRP5 showed a significant negative correlation with poor prognosis; each doubling of SFRP5 levels was associated with a 31% reduction in risk, highlighting the critical regulatory role of SFRP5 in the context of glucose metabolism disorders [39]. Under hyperglycemic conditions, decreased SFRP5 expression exacerbates ceramide accumulation, potentially triggering axonal degeneration through activation of the protein kinase C (PKC) pathway [40]. Axonal degeneration is a core pathological event in ophthalmic diseases such as diabetic retinopathy and glaucoma [41, 42], suggesting that SFRP5 may also participate in hyperglycemia‐induced nerve damage by regulating axonal homeostasis in retinal ganglion cells. Additionally, a decrease in serum SFRP5 levels during the first trimester precedes clinical elevations in blood glucose, suggesting that SFRP5 may represent an early event in the disruption of glucose homeostasis and could serve as a sensitive biomarker for glucose metabolism disorders [43].

Animal experiments have further elucidated the molecular basis of SFRP5 in regulating glucose metabolism. In obese and prediabetic mice, overexpression of SFRP5 exacerbated hyperglycemia and glucose intolerance, whereas treatment with an anti‐SFRP5 monoclonal antibody improved these phenotypes, suggesting that under these pathological conditions, SFRP5 acts as a negative regulator of glucose metabolism [44]. Notably, population studies show that decreased SFRP5 levels are associated with elevated blood glucose, whereas animal experiments indicate that SFRP5 overexpression exacerbates hyperglycemia. These seemingly contradictory findings precisely suggest that, as a regulator of glucose homeostasis, SFRP5 levels must be maintained within an appropriate range, as either excessively high or low levels may disrupt glucose balance. Therefore, SFRP5 is not simply a protective or deleterious factor but rather a key regulatory molecule that maintains glucose homeostasis.

### 4.2 SFRP5‐Mediated Regulation of Lipid Metabolism

SFRP5 exerts a dual regulatory role in lipid metabolism by inhibiting Wnt signaling pathways. On one hand, SFRP5 inhibits the canonical Wnt/β‐catenin signaling pathway, relieving its suppression of lipogenesis, promoting the differentiation of preadipocytes into mature adipocytes, and thereby increasing the lipid storage capacity of adipose tissue. On the other hand, SFRP5 suppresses Wnt signaling in adipocytes, downregulating the activity of mitochondrial function regulators such as *PGC1α* and mitochondrial transcription factor A, thereby inhibiting mitochondrial oxidative metabolism, reducing oxygen consumption, and promoting lipid storage and cellular hypertrophy [45]. This dual action both increases the lipid storage capacity of adipocytes and reduces their own energy expenditure, forming an efficient lipid metabolism pattern that maintains systemic metabolic homeostasis under conditions of energy excess.

Clinical studies have found that SFRP5 mRNA expression levels are significantly elevated in the adipose tissue of obese children and adolescents. After 1 year of lifestyle intervention, SFRP5 levels decreased, and this change was correlated with metabolic improvement, suggesting that its expression level is closely related to obesity severity [46]. Furthermore, in elderly populations, low SFRP5 levels are significantly associated with increased arterial stiffness, suggesting that decreased SFRP5 levels may lead to lipid metabolism disorders and subsequent impairment of vascular function. Animal experiments further revealed that SFRP5 knockout mice exhibit resistance to diet‐induced obesity. The mechanism lies in the fact that SFRP5 deficiency relieves inhibition of Wnt signaling, leading to enhanced mitochondrial oxidative metabolism and increased oxygen consumption in adipocytes, causing ingested energy to be more readily utilized for consumption rather than storage, thereby diminishing lipid accumulation capacity. This finding confirms that SFRP5 plays a critical regulatory role in adipose tissue energy metabolism and lipid storage by inhibiting Wnt signaling pathways.

In addition, SFRP5 also exerts important effects on hepatic lipid metabolism. In a mouse model of microcystin‐LR‐induced hepatic lipid metabolism disorder, SFRP5 expression was decreased, whereas overexpression of SFRP5 alleviated hepatic inflammation and lipid metabolic abnormalities [47]. In patients with non‐alcoholic fatty liver disease, hepatic SFRP5 expression was compensatorily elevated during the simple steatosis stage but downregulated upon progression to non‐alcoholic steatohepatitis [48]. This dynamic change suggests that SFRP5 may exert a protective role in the early stages of fatty liver disease but becomes dysfunctional in the advanced stages.

**Figure 4:**
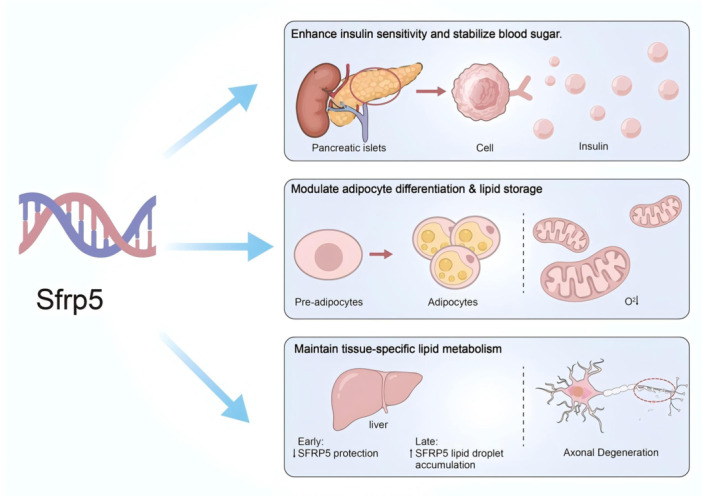
Regulatory mechanisms of SFRP5 in glucose and lipid metabolism.

The above evidence collectively corroborates the core function of SFRP5 in promoting lipid storage from both positive and negative perspectives. Compensatory elevation of SFRP5 expression in the adipose tissue of obese children represents an adaptive response to expand lipid storage capacity. In SFRP5 knockout mice, relief of Wnt signaling inhibition enhances mitochondrial oxidative metabolism, thereby conferring resistance to obesity. The underlying mechanism is that SFRP5, on one hand, promotes adipocyte differentiation to increase lipid storage capacity, and on the other hand, inhibits mitochondrial oxidative metabolism to reduce energy expenditure. The dynamic change of SFRP5 expression, initially increasing and then decreasing in non‐alcoholic fatty liver disease, further corroborates its regulatory role in response to changes in lipid status. In summary, SFRP5 integrates glucose and lipid metabolism to function as a key molecule in maintaining energy homeostasis. Its levels must be maintained within an appropriate range, as either excessively high or low levels may disrupt energy balance. This characteristic endows SFRP5 with potential as a biomarker for metabolic diseases and as a target for therapeutic intervention. The regulatory mechanisms of SFRP5 in glucose and lipid metabolism are illustrated in Figure 4.

## 5 Multiple Roles of SFRP5 in the Nervous System: From Development to Protection

### 5.1 SFRP5 and Neurodevelopment

Recent studies have shown that SFRP5 is widely expressed in the nervous system and participates in the regulation of neurogenesis and synaptogenesis.

Neurogenesis is the process by which neural stem cells proliferate and differentiate to generate new neurons, which is crucial for proper brain development and the establishment of functional connections in mammals [49, 50, 51]. Georgios Kalamakis and colleagues, using the Markov Chain Computational Method (MCCM) to analyze single‐cell transcriptomic data of neural stem cells [52], identified Sfrp5 as one of the genes specifically highly expressed in activated neural stem cells (aNSCs) of aged mice. Their team further conducted in vivo experiments in mice using anti‐SFRP5 neutralizing antibodies and found that inhibiting SFRP5 significantly reduced the number of label‐retaining cells (LRCs) in the brains of aged mice, suggesting that SFRP5 plays an important role in maintaining neural stem cell homeostasis.

Synaptogenesis forms the foundation of neural network construction and is essential for higher brain functions such as learning and memory [53, 54]. The Wnt signaling pathway plays an important role in synaptogenesis within the central nervous system. Studies have shown that Wnt7a induces the aggregation of synaptic proteins and promotes the formation of cerebellar glomerular synapses, whereas its mutant mice exhibit delayed synaptogenesis [55]. In addition, Wnt signaling promotes axonal remodeling and the recruitment of synaptic proteins, increases the expression of the presynaptic membrane protein synapsin 1, and influences synaptogenesis and normal function [56]. As a natural antagonist of Wnt signaling, we hypothesize that SFRP5 may participate in the fine regulation of synaptic plasticity by modulating Wnt signaling and thereby affecting the expression of molecules related to synaptic plasticity.

Notably, previous findings from our team are highly consistent with this mechanism [57]. In a VPA‐induced autism model rat, we observed that SFRP5 protein expression in the hippocampus was significantly elevated compared to the normal group, accompanied by significant inhibition of Wnt pathway proteins, while its expression level was markedly restored after acupuncture intervention. Meanwhile, transmission electron microscopy results showed that in the model group, the presynaptic and postsynaptic membrane structures of hippocampal neurons were blurred or even disrupted, and the number of synaptic vesicles was reduced with obvious damage. Following acupuncture intervention, the synaptic ultrastructural damage was significantly improved, manifested as clearer synaptic membrane structures and an increased number of synaptic vesicles. Considering the regulatory role of SFRP5 in neurogenesis and synaptogenesis described above, we hypothesize that acupuncture may influence synaptic structural remodeling by modulating the SFRP5‐mediated Wnt signaling pathway, thereby improving the behavioral performance of autism model rats. This finding provides new experimental evidence for further understanding the function of SFRP5 in neurodevelopment and its potential as a target for acupuncture therapy.

### 5.2 Regulation of the Neuroimmune Microenvironment by SFRP5

Microglia are key immune regulators in the central nervous system, and their polarization state plays a critical role in inflammatory responses and repair processes [58]. Previous studies have shown that M1 microglia are primarily involved in pro‐inflammatory responses, while M2 microglia participate in anti‐inflammatory effects and tissue repair [59, 60]. Wnt5a is a key factor regulating microglial polarization, inducing the transition of microglia toward the pro‐inflammatory M1 phenotype. In the M1 polarized state, microglia release various pro‐inflammatory cytokines such as tumor necrosis factor‐α (TNF‐α), interleukin‐6 (IL‐6), and interleukin‐1β (IL‐1β). These inflammatory factors not only directly inhibit the proliferation and differentiation of neural stem cells but also interfere with the normal process of synaptic pruning, thereby affecting the precise establishment of neural circuits. As an endogenous antagonist of Wnt5a, SFRP5 competitively binds Wnt5a, effectively inhibiting excessive microglial activation, alleviating neuroinflammation, and providing a favorable microenvironment for neurogenesis.

Recent studies have further elucidated the key role of the Wnt5a signaling pathway in the immune regulation of microglia. In a study by Ji Chen and colleagues [61], by constructing a targeted neural stem cell‐derived extracellular vesicle delivery system (ZH‐1c‐EVs@SIN), they found that this nanomedicine effectively inhibited the WNT5a signaling pathway, promoted the transition of microglia from the pro‐inflammatory M1 phenotype to the anti‐inflammatory M2 phenotype, significantly reduced the expression of inflammatory factors, and restored the levels of neuregulin. This intervention strategy significantly improved pain‐related behaviors and the histopathological manifestations of nerve injury. This study not only confirms the central role of the Wnt5a signaling pathway in regulating microglial polarization but also provides a new strategy for targeting this pathway in the treatment of neuroinflammatory diseases.

Astrocytes also participate in the regulation of neuroinflammation under metabolic stress and serve as one of the important sources of Wnt5a in the central nervous system. Halleskog and colleagues, using immunohistochemical techniques, demonstrated for the first time [62] that astrocytes in the adult mouse brain highly express Wnt5a protein, providing a molecular basis for astrocyte‐microglia signal communication. The study further found that astrocyte‐derived Wnt5a acts on adjacent microglia, inducing their pro‐inflammatory transformation. This is specifically manifested by the upregulated expression of inducible nitric oxide synthase (iNOS), cyclooxygenase‐2 (COX‐2), various cytokines, and chemokines, collectively amplifying neuroinflammatory responses. As an endogenous antagonist of Wnt5a, SFRP5 competitively binds Wnt5a, blocking its activation of astrocytes and microglia, thereby inhibiting the cascade amplification effect of neuroinflammation and maintaining neuronal functional stability.

### 5.3 Role of SFRP5 in the Peripheral Nervous System

In the peripheral nervous system, SFRP5 influences peripheral nerve function by regulating the neural lipid microenvironment. Peripheral nerve injury is a common condition that has long been a focus of neuroscience research. Lipid metabolism abnormalities have been recognized as a core pathological feature of both hereditary and acquired peripheral neuropathies (PNs) [63].

**Figure 5:**
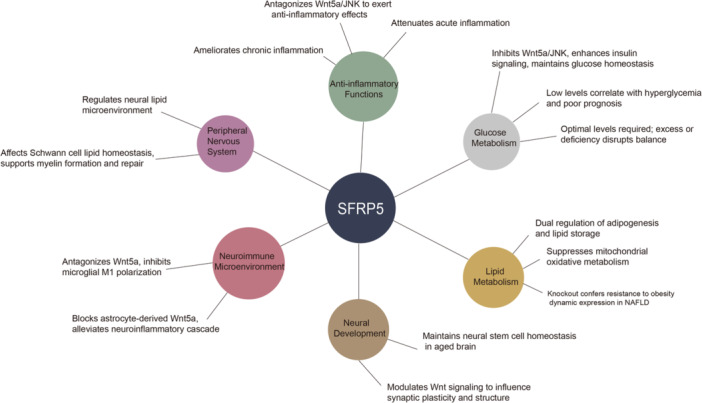
Multiple biological functions of SFRP5.

Following peripheral nerve injury, Wallerian degeneration is a characteristic pathological stage in the nerve repair process, during which Schwann cells engage in complex interactions with various surrounding cells. After necrotic axons and myelin fragments are broken down and phagocytosed by macrophages, Schwann cells proliferate to form channels that guide regenerating axons into the distal stump [64]. Wnt5a significantly influences the proliferation and regenerative capacity of Schwann cells through non‐canonical signaling pathways [65]. Studies have shown that silencing the Wnt5a gene leads to a significant increase in the expression of myelin‐associated glycoprotein (MAG) in Schwann cells. MAG, as an important myelin component protein, may inhibit axonal regeneration and disrupt myelin lipid homeostasis when aberrantly overexpressed [66]. Given that decreased SFRP5 expression leads to excessive activation of Wnt5a signaling, we hypothesize that this may disrupt lipid homeostasis in Schwann cells, thereby affecting myelin formation and maintenance. The multiple biological functions of SFRP5 in anti‐inflammation, energy metabolism, and the nervous system are shown in Figure 5, and the recent research progress is summarized in Table 1.

**Table 1:** Research progress on SFRP5 in recent years.

	Subject	Discovery	Reference
Anti‐inflammatory Functions of SFRP5	Human	SFRP5 levels negatively correlate with sepsis severity and recover after treatment	[27]
	Human	Serum SFRP5 levels are decreased in patients with severe periodontitis	[29]
	Mouse	Local administration of recombinant SFRP5 reduces gingival inflammation and alveolar bone loss.	[30]
	Mouse	Intra‐articular injection of recombinant SFRP5 alleviates cartilage degeneration.	[33]
	Cell	Overexpression of SFRP5 inhibits pro‐inflammatory cytokine production in rheumatoid arthritis synoviocytes.	[34]
SFRP5 in the Regulation of Glucose Metabolism	Human	Low SFRP5 level is an independent risk factor for elevated fasting glucose in adolescents.	[37]
	Human	SFRP5 levels negatively correlate with fasting glucose in the elderly	[38]
	Human	SFRP5 levels negatively correlate with poor prognosis in heart failure patients with T2DM	[39]
	Human	Decreased first‐trimester SFRP5 levels predict GDM risk	[43]
	Mouse	Overexpression of SFRP5 exacerbates hyperglycemia, while anti‐SFRP5 antibody improves glucose metabolism.	[44]
SFRP5 in the Regulation of Lipid Metabolism	Human	SFRP5 expression in adipose tissue is elevated in obese children and decreases after lifestyle intervention	[46]
	Human	Low SFRP5 levels are associated with increased arterial stiffness in the elderly.	[38]
	Mouse	Overexpression of SFRP5 alleviates hepatic inflammation and lipid metabolism disorders.	[47]
	Human	SFRP5 expression is compensatorily increased in simple steatosis but downregulated in NASH	[48]
SFRP5 and Neural Development	Mouse	Sfrp5 is highly expressed in activated neural stem cells of aged mice; neutralizing SFRP5 reduces LRCs	[52]
	Rat	Acupuncture restores SFRP5 expression and improves synaptic structural damage in the autism model.	[57]
SFRP5 in the Neuroimmune Microenvironment	Cell/Mouse	Targeting the WNT5a pathway promotes microglial M1‐to‐M2 transition and alleviates neuroinflammation.	[61]
	Cell	Astrocyte‐derived Wnt5a induces pro‐inflammatory transformation in microglia.	[62]
SFRP5 in the Peripheral Nervous System	Cell	Wnt5a silencing upregulates MAG expression, potentially inhibiting axonal regeneration.	[66]

Abbreviations: GDM, gestational diabetes mellitus; LRCs, label‐retaining cells; MAG, myelin‐associated glycoprotein; NASH, nonalcoholic steatohepatitis; SFRP5, secreted frizzled‐related protein 5; T2DM, type 2 diabetes mellitus; WNT5a, Wingless‐type MMTV integration site family member 5A.

## 6 Pathological Mechanisms of the SFRP5‐Lipid Metabolism Axis in Neural Development

### 6.1 Association Between Metabolic Disorders and Neural Development

Lipid metabolism plays a critical role in the nervous system. Lipid droplets (LDs), as the primary organelles for neutral lipid storage, are widely present in neurons and glial cells, originating from the synthesis of triglycerides (TGs) and cholesterol esters (CEs) in the endoplasmic reticulum [67, 68]. Herker et al. found that intracellular lipid homeostasis participates in maintaining mitochondrial function and reducing reactive oxygen species (ROS) accumulation, exerting important effects through mechanisms such as regulating energy supply, counteracting oxidative stress, and maintaining membrane structural stability [69]. Excessive free fatty acids (FFAs), if not promptly esterified and stored in LDs, can lead to lipotoxicity, resulting in mitochondrial dysfunction, endoplasmic reticulum stress, and oxidative damage [70]. In animal studies on Alzheimer's disease (AD), Hamilton et al. discovered that abnormal accumulation of LDs is closely associated with amyloid plaques and α‐synuclein aggregation, leading to cognitive decline [71]. Additionally, ROS generated from lipid peroxidation can further disrupt neuronal membrane integrity, exacerbating neuroinflammation and apoptosis [72].

Accumulating evidence indicates a significant association between metabolic disorders and adverse neurodevelopmental outcomes. Maternal obesity and diabetes during pregnancy are closely linked to an increased risk of autism spectrum disorder (ASD), attention‐deficit/hyperactivity disorder, and intellectual disability in offspring. High‐fat and high‐sugar diets not only alter maternal metabolic status but also affect placental function and the fetal developmental environment, interfering with normal fetal nervous system development. Numerous epidemiological studies have identified maternal metabolic disorders as significant risk factors for adverse neurodevelopmental outcomes in offspring [73]. Among mothers with overweight and obesity, each standard deviation decrease in postpartum low‐density lipoprotein (LDL) concentration was associated with a 54% increased risk of ASD in offspring. Furthermore, mothers in the lowest LDL group had an approximately 4.6‐fold higher risk of ASD in their offspring compared to those in the highest group [74]. A multicenter study by Windham et al. further explored the association between preconception body weight and gestational weight gain with offspring neurodevelopment [75]. The results indicated a positive correlation between maternal preconception obesity and ASD risk in offspring. Notably, higher gestational weight gain (GWG) showed an even stronger association with ASD risk, with the highest quintile group exhibiting a 58% increased risk compared to the middle group. This suggests that maternal lipid metabolism disorders may increase the risk of ASD in offspring by interfering with cholesterol supply during fetal neural development, providing new insights into potential pathogenic factors for neurodevelopmental abnormalities.

In this process, SFRP5, as a key molecule linking metabolic status and neurodevelopment, may mediate this risk through changes in its expression and function. Animal studies have shown that a high‐fat diet significantly reduces SFRP5 expression in the hypothalamus while increasing hypothalamic inflammation and oxidative stress [76]. In this study, C57BL/6 pregnant mice received a high‐fat diet intervention from gestational day 1, while the control group was fed a normal diet. The results showed that compared to the control group, the offspring in the high‐fat diet group exhibited a significant 42% reduction in SFRP5 mRNA expression in the hypothalamus, with protein expression levels also decreased by 38%. Meanwhile, the levels of inflammatory cytokines TNF‐α and IL‐6 in the hypothalamic tissue of the high‐fat diet offspring were significantly elevated, and the content of the oxidative stress marker malondialdehyde (MDA) increased by 1.7‐fold. During critical developmental periods, such changes may lead to persistent neurological dysfunction by altering neurogenesis, synaptogenesis, and neural network establishment.

### 6.2 Signaling Pathway Interactions and Neurodevelopment

As a member of the secreted frizzled‐related protein family, SFRP5 not only antagonizes the binding of Wnt proteins to Frizzled receptors through its CRD domain, thereby regulating the canonical Wnt/β‐catenin pathway, but also participates extensively in non‐Wnt‐dependent signaling pathways, forming a complex regulatory network. Studies have shown that SFRP5 is involved in regulating the activity of the Hedgehog (Hh) signaling pathway [77]. As a key morphogen governing dorsoventral patterning of the neural tube, the gradient distribution of Hh signaling directly determines the fate specification of motor neurons and interneurons. Recent optogenetic studies have demonstrated that the concentration gradient and exposure duration of Shh collectively regulate the differentiation of ventral neural tube cell fates [78]. SFRP5 may indirectly influence the establishment of neural tube patterning by modulating the stability of Hh ligands or their interaction with the receptor Ptch1.

Furthermore, the role of SFRP5 in metabolic regulation and neurotrophic factor signaling pathways is gaining increasing attention. Studies have shown that metabolic stress, such as a high‐fat diet, suppresses the expression of brain‐derived neurotrophic factor (BDNF) in the brain, thereby impairing synaptic plasticity and leading to cognitive decline [79]. Notably, SFRP5, as an adipokine with anti‐inflammatory properties, may indirectly upregulate the expression and secretion of BDNF by improving the metabolic microenvironment in the peripheral and central nervous systems and suppressing the Wnt5a/JNK inflammatory pathway in adipose tissue [80]. BDNF, in turn, activates its specific receptor TrkB, thereby exerting important effects in supporting neuronal survival, promoting differentiation, and maintaining synaptic function.

### 6.3 Impact of SFRP5‐Lipid Metabolism Imbalance on Neurodevelopment

**Figure 6:**
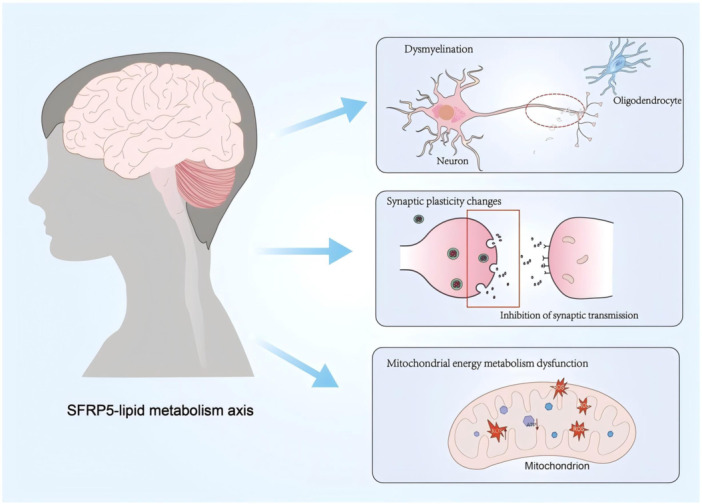
Pathological mechanisms of the SFRP5‐lipid metabolism axis in neural development.

Abnormal SFRP5 expression may interfere with neurodevelopmental processes through multiple mechanisms (as shown in Figure 6):

(1) Myelination impairment: Downregulation of SFRP5 expression can induce lipid metabolism disorders in oligodendrocytes, affecting the biosynthesis of cholesterol and sphingomyelin. These two lipids are critical components of myelin structure and function, and their metabolic abnormalities directly impair the proper formation and stability of myelin sheaths. Myelin abnormalities further lead to decreased nerve impulse conduction velocity and impaired neural network synchronization, which are closely associated with cognitive deficits and behavioral abnormalities commonly observed in adverse neurodevelopmental outcomes [81]. Additionally, SFRP5 may influence the differentiation process of oligodendrocyte precursor cells (OPCs) into mature oligodendrocytes by affecting Wnt signaling pathways, thereby impairing myelin repair and regeneration capacity.

(2) Synaptic plasticity alterations: Wnt signaling pathways are involved in regulating synaptogenesis and plasticity. As a key regulator of Wnt signaling pathways, abnormal SFRP5 expression leads to dysregulated Wnt signaling, affecting synaptic protein synthesis, dendritic spine morphology, and neurotransmitter receptor trafficking, thereby altering synaptic function. Studies have shown that aberrant elevation of Wnt5a impairs hippocampal long‐term potentiation (LTP) and affects learning and memory function [82].

(3) Mitochondrial energy metabolism dysregulation: Abnormal SFRP5 expression may induce lipid metabolism disorders, leading to impaired β‐oxidation of long‐chain fatty acids within mitochondria, increased reactive oxygen species (ROS) generation, and reduced ATP synthesis efficiency, resulting in insufficient energy supply to neurons. Individuals with neurodevelopmental abnormalities often exhibit mitochondrial dysfunction [83, 84], and the SFRP5‐Wnt5a signaling axis may influence mitochondrial biogenesis and oxidative metabolic function. Furthermore, SFRP5 deficiency may relieve inhibition of the Wnt/JNK pathway, exacerbating oxidative stress and neuroinflammation, further disrupting neuronal energy homeostasis and ultimately leading to synaptic transmission deficits and neural network dysfunction.

(4) Retinal vascular developmental abnormalities: Wnt signaling pathways play critical roles in ocular vascular morphogenesis, participating in processes such as hyaloid vessel regression and retinal vascular network formation [85]. Loss‐of‐function mutations in Wnt signaling components can lead to hereditary ocular diseases such as Norrie disease and familial exudative vitreoretinopathy, whereas aberrantly increased Wnt signaling may be closely associated with pathological ocular neovascularization, such as in retinopathy of prematurity [86]. As a natural antagonist of Wnt signaling, abnormal SFRP5 expression may interfere with Wnt signaling homeostasis, affecting normal retinal vascular development and maintenance, thereby contributing to the visual complications associated with neurodevelopmental abnormalities [87].

## 7 Therapeutic Potential and Future Directions of SFRP5

### 7.1 SFRP5‐Based Therapeutic Strategies

Given the important roles of SFRP5 in metabolism and neuroprotection, enhancing SFRP5 signaling has emerged as a potential therapeutic strategy for various diseases:

(1) Recombinant SFRP5 protein therapy: In diseases characterized by reduced SFRP5 expression, such as diabetic peripheral neuropathy, supplementation with exogenous SFRP5 may restore its physiological function. Systemic administration of recombinant SFRP5 protein can neutralize overactive Wnt5a signaling, reduce inflammatory responses and insulin resistance, thereby improving nerve conduction function and slowing neuropathy progression. Conversely, in diseases characterized by SFRP5 overexpression, such as age‐related neuroregenerative disorders, the use of SFRP5‐specific antibodies can effectively block its binding to Wnt ligands, relieve inhibition of Wnt signaling pathways, and promote neural stem cell activation and injury repair [88]. However, the poor permeability of protein macromolecules across the blood‐brain barrier remains a major challenge for the treatment of central nervous system diseases [89, 90, 91]. Developing efficient central delivery systems, such as nanoparticle carriers and exosome encapsulation technologies, is critical for advancing clinical translation.

(2) Small molecule activators: Small molecule compounds offer unique advantages due to their favorable pharmacokinetic properties. Small molecules identified through high‐throughput screening can promote endogenous SFRP5 expression or enhance its binding affinity to Wnt5a. Notably, some classic drugs such as the insulin sensitizers rosiglitazone and metformin have been shown to significantly upregulate SFRP5 expression in adipose tissue and muscle through activation of pathways such as PPARγ, which may represent one of the mechanisms underlying their beneficial effects on metabolism and neural function.

(3) Gene therapy: Viral vector‐based gene therapy offers a long‐term solution for modulating SFRP5 expression. Using tissue‐specific promoters, recombinant adeno‐associated virus (AAV) serotypes can mediate long‐term, stable expression of the SFRP5 gene in the central nervous system. This approach holds significant promise for treating chronic metabolic diseases and neurodegenerative disorders. In particular, targeted delivery of the SFRP5 gene to energy metabolism centers may exert broad and sustained protective effects against obesity, type 2 diabetes, and their neurological complications by regulating systemic energy homeostasis and insulin sensitivity.

(4) Lifestyle interventions: Caloric restriction and regular aerobic exercise have been shown by multiple studies to significantly upregulate SFRP5 expression levels in white adipose tissue and the hypothalamus, which is likely closely associated with their ability to ameliorate low‐grade inflammation and improve insulin sensitivity. More importantly, early life represents a critical window for metabolic programming. Implementing scientific nutritional interventions during pregnancy and lactation may also reduce the risk of adverse neurodevelopmental outcomes in offspring by influencing SFRP5 expression levels, offering a highly promising public health strategy for early disease prevention.

(5) Acupuncture intervention: Acupuncture, as a safe and non‐invasive non‐pharmacological intervention, demonstrates unique potential in modulating the SFRP5 signaling pathway. Previous work from our team has shown that acupuncture may exert neuroprotective effects by restoring the balance of the SFRP5/Wnt signaling pathway. Compared with pharmacological interventions, acupuncture offers advantages such as multi‐target regulation and minimal side effects, making it particularly suitable for interventions in developmental central nervous system disorders.

### 7.2 Future Research Directions

Although significant progress has been made in recent years regarding the role of SFRP5 in lipid metabolism and neurodevelopment, the spatiotemporal specificity of its expression and function across different neurodevelopmental stages, brain regions, and specific cell types remains largely unexplored. Critically, as an antagonist of Wnt signaling pathways, the ultimate effects of SFRP5 may be highly dependent on developmental stage, cellular microenvironment, and interactions with other signaling pathways. Therefore, future research should employ single‐cell transcriptomics, spatial transcriptomics, and cell type‐specific genetic manipulation techniques to systematically dissect the dynamic regulatory networks of SFRP5 during neurogenesis, synaptogenesis, and myelination at single‐cell resolution, thereby elucidating the cellular heterogeneity and spatiotemporal specificity of its actions.

Secondly, the role of interactions between SFRP5 gene polymorphisms and environmental factors in susceptibility to adverse neurodevelopmental outcomes warrants further investigation. How such genetic variations synergize with environmental exposures, such as maternal metabolic disorders and perinatal inflammation, to regulate neurodevelopmental outcomes in offspring remains poorly understood. There is an urgent need for large‐scale prospective cohort epidemiological studies combined with multimodal epigenetic analyses to elucidate the transgenerational mechanisms by which metabolic stress during pregnancy influences SFRP5 gene methylation modifications and expression regulation in offspring.

Furthermore, the network regulatory mechanisms of the SFRP5‐mediated metabolism‐neural axis in neural development and related abnormalities urgently require elucidation through integrative multi‐omics approaches. Analyses at a single level are no longer sufficient to comprehensively reveal the central role of SFRP5 in lipid metabolism reprogramming, neuroimmune interactions, and mitochondrial energy metabolism. Future research should integrate genomic, epigenomic, proteomic, and metabolomic data to construct systems biology models of SFRP5‐related signaling pathways, thereby identifying key nodes amenable to intervention and providing theoretical foundations for precise targeted therapy.

In terms of translational research on therapeutic strategies, how to efficiently and safely deliver SFRP5‐targeting agents to the central nervous system remains a core technical bottleneck. The poor permeability of protein macromolecules across the blood‐brain barrier limits the application of recombinant SFRP5 protein in neurological disorders. Developing brain‐targeting nanodelivery systems, exosome carriers, or adeno‐associated virus (AAV)‐based gene therapy strategies will be critical for advancing SFRP5‐related interventions toward clinical translation. Additionally, the mechanisms by which non‐pharmacological interventions such as acupuncture regulate SFRP5 signaling pathways warrant further investigation. Future studies should combine neural circuit tracing, chemogenetics, and optogenetics to elucidate the specific neural circuits and molecular basis underlying acupuncture‐mediated regulation of SFRP5 signaling, thereby providing stronger theoretical support for its use as an adjunctive therapy for neural developmental abnormalities.

## 8 Conclusions

This article systematically reviews the multifaceted functions of SFRP5 as a key negative regulator of the Wnt signaling pathway, identifying its core roles in anti‐inflammatory regulation by inhibiting excessive activation of macrophages and microglia, in energy metabolism by dynamically maintaining insulin sensitivity and lipid homeostasis, and in nervous system development by regulating neural stem cell homeostasis, synaptic plasticity, and myelination. Aberrant SFRP5 expression may contribute to neural developmental abnormalities, with its potential relevance to conditions such as autism spectrum disorder suggested by immune‐metabolic imbalance via mechanisms including myelination impairment, altered synaptic plasticity, and mitochondrial energy metabolism dysregulation. Intervention strategies targeting the SFRP5/Wnt5a signaling pathway exhibit dual potential for anti‐inflammatory and metabolic regulation, with approaches such as recombinant protein therapy, small molecule activators, and gene therapy demonstrating broad prospects for clinical application. Future research should focus on elucidating its spatiotemporal expression specificity, mechanisms of action in specific immune cell subsets, and the development of efficient central delivery systems, thereby providing new targets for the precise prevention and treatment of neural developmental abnormalities.

## Author Contributions

**Qianfang Jia:** conceptualization, methodology. **Zihan Li:** conceptualization, methodology. **Tianyi Yang:** data curation, investigation. **Weihua Yang:** data curation, validation. **Wei Chi:** formal analysis, investigation.

## Conflicts of Interest

The authors declare no conflicts of interest.

## Data Availability

The authors have nothing to report.
